# Susceptibility to intestinal infection and diarrhoea in Zambian adults in relation to HIV status and CD4 count

**DOI:** 10.1186/1471-230X-9-7

**Published:** 2009-01-22

**Authors:** Paul Kelly, Jim Todd, Sandie Sianongo, James Mwansa, Henry Sinsungwe, Max Katubulushi, Michael J Farthing, Roger A Feldman

**Affiliations:** 1 Tropical Gastroenterology & Nutrition group, Department of Medicine, University of Zambia School of Medicine, Lusaka, Zambia; 2 Institute of Cell and Molecular Science, Barts & The London School of Medicine, Queen Mary University of London, UK; 3 Department of Infectious and Tropical Diseases, London School of Hygiene and Tropical Medicine, London, UK; 4 Sussex University, Brighton, UK

## Abstract

**Background:**

The HIV epidemic in sub-Saharan Africa has had a major impact on infectious disease, and there is currently great interest in the impact of HIV on intestinal barrier function. A three year longitudinal cohort study in a shanty compound in Lusaka, Zambia, carried out before anti-retroviral therapy was widely available, was used to assess the impact of HIV on susceptibility to intestinal infectious disease. We measured the incidence and seasonality of intestinal infection and diarrhoea, aggregation of disease in susceptible individuals, clustering by co-habitation and genetic relatedness, and the disease-to-infection ratio.

**Methods:**

Adults living in a small section of Misisi, Lusaka, were interviewed every two weeks to ascertain the incidence of diarrhoea. Monthly stool samples were analysed for selected pathogens. HIV status and CD4 count were determined annually.

**Results:**

HIV seroprevalence was 31% and the prevalence of immunosuppression (CD4 count 200 cells/μL or less) was 10%. Diarrhoea incidence was 1.1 episodes per year and the Incidence Rate Ratio for HIV infection was 2.4 (95%CI 1.7–3.3; p < 0.001). The disease-to-infection ratio was increased at all stages of HIV infection. Aggregation of diarrhoea in susceptible individuals was observed irrespective of immunosuppression, but there was little evidence of clustering by co-habitation or genetic relatedness. There was no evidence of aggregation of asymptomatic infections.

**Conclusion:**

HIV has an impact on intestinal infection at all stages, with an increased disease-to-infection ratio. The aggregation of disease in susceptible individuals irrespective of CD4 count suggests that this phenomenon is not a function of cell mediated immunity.

## Background

Intestinal infection causing diarrhoeal disease is a dominant contributor to high death rates and developmental retardation in developing countries [1]. Most of the morbidity and mortality due to diarrhoea is in young children, but in the wake of the HIV pandemic, diarrhoea and secondary malnutrition emerged as a major problem in adults too, especially in sub-Saharan Africa [2]. The epidemiology of intestinal infectious disease is complex, with over 30 viruses, bacteria or protozoa being implicated in its aetiology [3]. Although widespread uptake of highly active anti-retroviral therapy (HAART) in urban Zambia [4] has reduced the burden of diarrhoeal disease in adults despite the continuing high prevalence of HIV, people with AIDS-related diarrhoea still present to hospitals and clinics, and mortality in such cases is still high. Before the anti-retroviral era, and regrettably still in many parts of the continent, the HIV epidemic created populations in which a significant proportion of the community was immunosuppressed at any one time, but the implications of this degree of population immunosuppression for the impact of intestinal pathogens was not defined. It was never clear whether advanced immunosuppression makes intestinal disease inevitable in adults living in the most environmentally deprived conditions. Nor was it established whether in such populations the immunosuppression led to increased infection with diarrhoea-causing pathogens, or to increased disease manifestations in carriers of pathogens. Understanding alterations in risk of infection and risk of disease help understand the nature of the immunological defect.

We set up a longitudinal cohort study in an impoverished population in Misisi, Lusaka [5] in order to determine the incidence of intestinal infection and the proportion of such infections associated with diarrhoeal disease. As the cohort was fairly representative of a particular community, it was possible to analyse the phenomenon of aggregation. It is well known that intestinal helminth infections display aggregation [6-8] meaning that certain susceptible individuals carry large numbers of worms and therefore a disproportionate fraction of the burden of disease [9]. There is also some evidence that incidence of diarrhoeal disease is aggregated in some highly susceptible children [10,11] and adults [12] in an analogous way. In this paper we analyse diarrhoea and infection separately which allows us to demonstrate that the disease-to-infection ratio is elevated at all stages of HIV infection. We also show that diarrhoea incidence displays aggregation in both immunocompetent and immunosuppressed Zambian adults.

Recently there has been great interest in the impact of HIV on the gut [13], both because of virally-induced T cell depletion [14-16], and because intestinal damage may drive bacterial translocation and hence immune activation [17]. The recognition that the gut is an important target organ for HIV lends great urgency to an understanding of the functional change in immune function both in early and late stage disease. On balance, most morphometric [5,18-21] and functional [5,22-24] evidence suggests that intestinal damage (enteropathy) is largely a feature of late HIV disease. Our study design also allowed us to analyse the stage of HIV disease at which susceptibility to infection changes.

## Methods

### Study setting

A cohort study of diarrhoea and intestinal infection was carried out in Misisi compound, an unplanned residential area in the southern part of Lusaka which was illegal until 1995 and where sanitation is rudimentary, housing is of poor quality, and overcrowding is intense. Mortality rates in adults are high [25], probably attributable to the severity of the HIV epidemic. In Lusaka the HIV seroprevalence is probably static at around 22% [26]. Authorisation was obtained to study part of one sector of Misisi from the Lusaka Urban District Health Management Board, and approval was obtained from the Research Ethics Committees of the University of Zambia and the London School of Hygiene and Tropical Medicine. The study was set up in April 1999, and the cohort followed until May 2002. At this time anti-retroviral therapy and co-trimoxazole prophylaxis were not standard of care and not given to the study cohort.

The study included only residents in a defined area. Recruitment of a cohort within a cluster of closely grouped houses was felt to be important to maximise retention in the study and to increase the probability that residents would share a similar degree of exposure to enteric pathogens. Drinking water in this area is drawn from only 20 standpipes, and sanitation is provided by 25 pit latrines. Food is obtained from a small number of local markets. At the outset, a house to house survey was conducted to include all households in the study area, during which the purpose of the study was explained, and one respondent per household was interviewed to obtain a demographic profile. Every adult resident (18 years of age or more) was then invited to the nearest urban health centre, a 10 minute walk from the study area, for interview and examination. At this interview, the purpose of the study was explained in more detail and a two page information sheet read out in one of four local languages. This information included explicit description (including photographs) of the procedures involved in participating in the study over three years. The recruitment and consent process included information about HIV testing, and participants who consented to inclusion in the study were offered the option of HIV testing (together with CD4 count) with full pre- and post-test counselling, but they were also free not to have the test.

At the initial interview, questions were also asked about the source of drinking water habitually used, about how often and when drinking is boiled, about alternative sources of drinking water, and whether water drunk outside the home is boiled. Nutritional assessment included weight and height (combined into the body mass index in kg/m^2^) and serum retinol concentration measured by high performance liquid chromatography at the mid-point of the study as one of several potential markers of micronutrient status. At the mid-point of the study, the nurses were asked to score each household for hygiene facilities and practices in the home, as previously described [5]. A scale of 1–10 was used, up to 2 points being given for each of the following: overall cleanliness of the house, water storage facilities, food storage facilities, hand-washing facilities and their use, and sanitation facilities.

Each participant was interviewed every two weeks using a standardised questionnaire and asked to report any episodes of diarrhoea in the previous two weeks. Two definitions of diarrhoea were used: either a subjective report of symptoms as previously described [27], or the presence of increased water in stool samples. They also provided a stool sample at some point during each month if there were no symptoms, or whenever there was diarrhoea. Only one sample was analysed each month; if diarrhoea supervened during the month and an 'asymptomatic' sample had already been submitted the diarrhoea sample result was used in its place. These samples were transported to the laboratory by 09.30 each day and processed immediately. Participants were also treated for any medical problems. Every year, subjects were counselled and offered an HIV test, and a CD4 count was performed irrespective of HIV status if consent was given.

In the laboratory, stool consistency (a measure of water content) was noted and protozoa and helminths identified in each sample by microscopy of a wet preparation, a Ziehl-Neelsen stained thin smear, and a very thin smear stained with Sianongo's modified trichrome stain [28]. Swabs which had been stabbed into Cary-Blair transport medium in the field clinic prior to transport to the hospital laboratory were used to inoculate MacConkey, xylose-lactose-sucrose, sorbitol-MacConkey, Yersinia selective and thiosulphate-citrate-bile salt agar plates. Colonies of non-lactose fermenting bacteria were subjected to biochemical characterisation using triple-sugar-iron, lysine-iron, sulphide-indole-motility, urea and Simmons citrate media, and interpreted using Cowan & Steel's manual [29]. Isolates picked at random were confirmed using the API 20E system. Micro-aerophilic conditions for isolation of *Campylobacter jejuni *were difficult to maintain and results for this organism are omitted.

In order to analyse the disease-to-infection ratio (i.e. the proportion of infection episodes which lead to diarrhoea), two definitions of intestinal infections were used. The restrictive definition included only infections associated with diarrhoea in this study (*C. parvum, I. belli*, microsporidia, *G. intestinalis, S. stercoralis, V. cholerae*). A more open definition included the first group together with other bacteria commonly associated with diarrhoea (*Salmonella *spp., *Shigella *spp., *A. hydrophila*).

### Data analysis

We analysed incidence of diarrhoea and of infection separately. For both diarrhoea and infection, each month was evaluated separately. Analysis of the records of diarrhoea month-by-month reveals that diarrhoea episodes of over 4 days duration could not have overlapped for more than 9 participants, and months of diarrhoea can therefore be regarded as separate episodes. Analysis with these participants excluded did not alter the results which include these participants. To determine whether incidence of intestinal infection could be distorted by persistent infections, the proportion of infectious episodes which could have overlapped into consecutive months was also analysed. At most, 11% of episodes of cryptosporidiosis, 10% of giardiasis and 1% of salmonellosis could have overlapped. Omission of these potentially non-incident episodes increased the disease-to-infection ratio from 18.4% to 18.8%, so we conclude that it is valid to consider each month as a discrete and independent period of observation.

The seasonality of diarrhoeal disease and of infections was assessed by grouping monthly data into six 2-month periods. Disease-to-infection ratio [30] was calculated as the proportion of months in which an intestinal infection was identified during which diarrhoea was reported. For the association between diarrhoea (defined either subjectively or by water content of stool samples) and pathogen infections, odds ratios (OR) were calculated and Mantel-Haenszel stratification used to adjust for HIV status. Fisher's exact and chi-squared tests were used to test the strength of these associations.

To analyse clustering of diarrhoea and infection, we excluded participants who had submitted less than 10 months of data. A negative binomial model was constructed and the value of alpha (a measure of aggregation) was used to describe clustering in susceptible individuals (i.e.aggregation). The model was then used (with a term to account for aggregation) to relate the incidence of diarrhoea to age, sex, HIV status, CD4 count below 200 cells/μL, body mass index below the median, serum retinol concentration below the median, water boiling practices, and the household hygiene index. The negative binomial model was also used to compare the effect of clustering between individuals, for household co-habitation (defined as eating together) and first-degree genetic relatedness (i.e. parent/child or sibling relationships). Data analysis was performed using STATA 8.2 (Stata Corp, College Station, Texas).

## Results

In April 1999 the initial house-to-house survey identified 506 adults as resident in the study area [31]. Of these, 270 (53%) consented to participate in the study, and 206 (76%, 41% of all residents) actively participated in the follow-up and are included in this analysis (Table 1). After one year, 174 (84%) remained, 148 (72%) after two years, and at the close of the third year, 121 (59%) were still included. By April 2002, 20 (9.7%) participants had died during the 3 years of follow-up. The majority of losses to follow up were due to moving house, usually to obtain better housing, to return to the family village, or to avoid rent arrears. Mean follow-up was 22.8 months.

**Table 1:** Demographic and clinical characteristics of 206 study participants

	Male	Female
n	70	136

Age (median, IQR)	38 (31–49)	31 (25–40)

Age (range)	21–74	18–79

HIV seropositive at baseline	14/60 (23%)	39/117 (33%)

Seroconverted to HIV during follow-up	2	4

CD4 count (cells/μl) if HIV positive (median, IQR) (range)	210 (125–432) (40–929)	299 (177–399) (51–810)

CD4 count (cells/μl) if HIV negative (median, IQR) (range)	742 (583–797) (269–1184)	812 (664–1001) (384–1623)

BMI (kg/m^2^) (median, IQR)	19.7 (18.1–21.5)	21.7 (19.9–24.6)

BMI below 18.5 kg/m^2^	25/75 (33%)	18/124 (15%)

Serum retinol (μmol/l) (median, IQR)	1.91 (1.52–2.41)	1.62 (1.30–2.08)

Hygiene score (median, IQR)	3 (2–5)	2 (2–6)

IQR, interquartile range; CD4, cluster differentiation antigen 4; HIV, human immunodeficiency virus; BMI, body mass index. *P *value refers to Kruskal-Wallis test of difference between medians, or Fisher's exact test of difference between proportions.

At recruitment, 177 of 206 participants underwent HIV testing, and 53 (30%) were seropositive (Table 1). A further 11 participants were tested later in the study and of these 5 were seropositive when tested, making a total of 58 (31%) positive of those tested at some point. Of these 58, 6 were incident infections. Overall, 17 (33%) of 52 HIV seropositive participants at recruitment had CD4 counts under 200 cells/μL (one test failed for technical reasons). This represents 10% (17/177) of all the adults in this population who were willing to participate. None of the HIV seronegative participants had CD4 counts below 200 cells/μL.

### Incidence of diarrhoea and intestinal infection

From the 206 subjects, a total of 5,199 person-months of observation were completed. Diarrhoea was reported in 488 months, and 4,711 months were diarrhoea-free, giving an overall incidence of 0.094 episodes per month or 1.1 episodes per year. In HIV seronegatives the rate was 0.068 per month and in HIV seropositives the rate was 0.153 per month.

A total of 4,780 stool samples were submitted for analysis, and results for bacteria, protozoa or helminths obtained in 4,731 (99%) samples. Mixed infections (with the pathogens defined above) were found in 12 samples. The presence of pathogens by stool consistency is shown in Table 2. Certain parasites (*I. butschlii, C. mesnili, A. lumbricoides*, and *E. nana*) were less frequently detected in more watery stools, consistent with a lack of pathogenicity. Infections with *C. parvum, I. belli*, and *G. intestinalis *were associated with increasing water content of the stool samples, and these associations were all stronger after Mantel-Haenszel stratification for HIV status (data not shown).

**Table 2:** Frequency of intestinal infection in relation to stool consistency

Organism	Formed	Soft	Loose	Watery	p	trend
	n = 2194	n = 2230	n = 290	n = 17		

*Cryptosporidium parvum*	16 (0.8)	34 (1.5)	7 (2.4)	0	0.02	0.004

*Isospora belli*	8 (0.4)	4 (0.2)	7 (2.4)	1 (5.9)	< 0.001	0.001

Microsporidia	3	3	1	0	ns	

*Giardia intestinalis*	16 (0.7)	33 (1.5)	9 (3.1)	2 (11.8)	< 0.001	< 0.001

*Blastocystis hominis*	144	150	30	1	ns	

*Entamoeba histolytica*	11	8	0	0	ns	

*Iodamoeba butschlii*	108 (4.9)	53 (2.4)	8 (2.8)	0	< 0.001	< 0.001

*Entamoeba hartmanii*	35	25	2	0	ns	

*Chilomastix mesnili*	173 (7.9)	138 (6.2)	6 (2.1)	0	0.001	< 0.001

*Ascaris lumbricoides*	383 (17.5)	326 (14.6)	37 (12.8)	0	0.007	0.001

Hookworm	76	63	7	1	ns	

*Strongyloides stercoralis*	4 (0.2)	8 (0.4)	3 (1)	0	0.1	0.04

*Schistosoma mansoni*	11	10	2	0	ns	

*Endolimax nana*	212 (9.7)	143 (6.4)	11 (3.8)	0	< 0.001	< 0.001

*Hymenolepis nana*	16	12	0	0	ns	

*Trichuris trichiura*	1	6	0	0	ns	

*Taenia saginata*	3	6	2	0	ns	

*Salmonella *spp.	24	31	3	0	ns	

*Shigella *spp.	9	7	2	0	ns	

*Aeromonas hydrophila*	9	8	0	0	ns	

*Citrobacter rodentium*	417	406	61	3	ns	

*Vibrio cholerae*	3	2	1	0	ns	

Percentages are shown in brackets where significant differences were found. 'P' was derived by Fisher's exact test across all groups, 'trend' refers to the P value using the χ^2 ^for trend test to detect progressive changes in frequency with trends in consistency.

For 4,294 (91%) samples the pathogen results were related to the HIV status of the individual. *C. parvum, I. belli*, and *C. rodentium *infections were significantly commoner in HIV-infected adults (Table 3). Certain non-pathogenic protozoa and helminths were less common in HIV-infected participants (Table 3), possibly due to self-treatment with antimicrobials and anti-helminthics.

**Table 3:** Frequency of intestinal infection in relation to HIV

Organism	HIV+	HIV-	*P*	HIV positive	HIV positive
	n = 1313	n = 2981		CD4 < 200 cells/μl	CD4 > 200 cells/μl

*C. parvum*	36 (2.7)	18 (0.6)	< 0.001	25	6

*I. belli*	13 (1.0)	7 (0.2)	0.002	8	1

*I. butschlii*	29 (2.2)	130 (4.4)	< 0.001	7	13

*C. mesnili*	69 (5.3)	220 (7.4)	0.01	26	29

*A. lumbricoides*	172 (13.1)	520 (17.4)	< 0.001	48	72

*S. stercoralis*	0	14 (0.5)	0.008	-	-

*C. rodentium*	265 (22.7)	465 (17.8)	0.001	65	138

Only infections which differ significantly with HIV status are shown. *P *values derived by Fisher's exact test.

**Figure 1:**
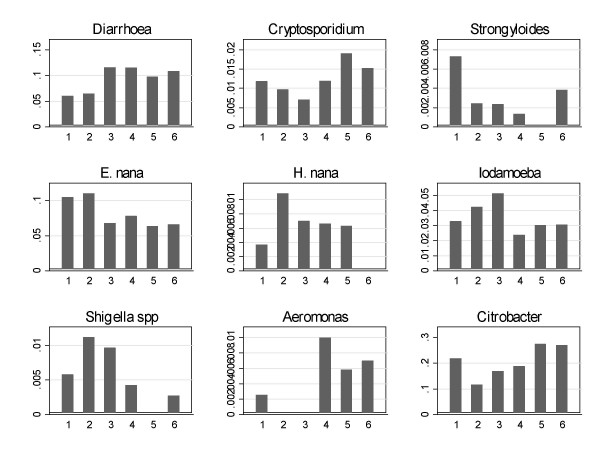
**Seasonal variation in diarrhoea and in eight intestinal infections**. The seasons (each of 2 months) are shown along the x axis (1 May, June; 2 July, August; 3 September, October; 4 November, December; 5 January, February; 6 March, April) and the incidence of that infection per 'season' on the y axis. Rainfall is November-March, so shigellosis is commoner in the dry season and aeromoniasis during the rains. Infections which are listed in Table 1 and not shown here did not display seasonality.

There was clear seasonal variation in the incidence both of diarrhoea and of some infections. The incidence of *Cryptosporidium parvum, Endolimax nana, Iodamoeba butschlii, Strongyloides stercoralis, Hymenolepis nana, Shigella *spp., *Aeromonas hydrophila *and *Citrobacter rodentium *infections was seasonal, but the pattern of seasonality differed (Fig 1). Infections with *Isospora belli, Giardia intestinalis, Blastocystis hominis, Ascaris lumbricoides*, hookworm, *Chilomastix mesnili*, and *Salmonella *spp. were not seasonal. The seasonal variation in diarrhoea incidence was evident in both HIV seropositive and seronegative participants.

### Association between infection and reported symptoms of diarrhoea

For 4,198 (89%) samples the pathogen results could be related to the presence or absence of diarrhoea reported within two weeks of the date submitted, and associations shown in Table 4. There were few infections which were clearly associated with diarrhoea, and the majority of infections detected, even with known pathogens, were asymptomatic. Infection with *I. belli*, microsporidia, or *V. cholerae *were significantly associated with diarrhoea after stratification by HIV status: the respective Mantel-Haenszel Odds Ratios were 5.9 (95% CI 2.0–16.8; p = 0.0002), 9.1 (1.6–53; p = 0.002) and 13 (1.8–94; p = 0.001).

**Table 4:** Frequency of intestinal infection in relation to recalled diarrhoea

Organism	Frequency (%) in months when diarrhoea reported n = 408	Frequency (%) in months when diarrhoea not reported n = 3790	*P*
*Cryptosporidium parvum*	11 (2.7)	38 (1.0)	0.006

*Isospora belli*	7 (1.7)	13 (0.3)	0.002

Microsporidia	3 (0.7)	5 (0.1)	0.035

*Giardia intestinalis*	7 (1.7)	47 (1.2)	ns

*Blastocystis hominis*	31 (7.6)	249 (6.6)	ns

*Entamoeba histolytica*	0	13 (0.3)	ns

*Iodamoeba butschlii*	13 (3.2)	142 (3.8)	ns

*Entamoeba hartmanii*	4 (1.0)	53 (1.4)	ns

*Chilomastix mesnili*	30 (7.3)	250 (6.6)	ns

*Ascaris lumbricoides*	68 (16.7)	585 (15.4)	ns

Hookworm	14 (3.4)	108 (2.9)	ns

*Strongyloides stercoralis*	1 (0.3)	12 (0.3)	ns

*Schistosoma mansoni*	2 (0.5)	16 (0.4)	ns

*Endolimax nana*	31 (7.6)	291 (7.7)	ns

*Hymenolepis nana*	1 (0.3)	23 (0.6)	ns

*Trichuris trichiura*	0	6 (0.2)	ns

*Taenia saginata*	0	8 (0.2)	ns

*Salmonella *spp.	5 (1.3)	46 (1.3)	ns

*Shigella *spp.	2 (0.5)	12 (0.3)	ns

*Aeromonas hydrophila*	3 (0.8)	13 (0.4)	ns

*Citrobacter rodentium*	86 (23.1)	786 (22.5)	ns

*Vibrio cholerae*	2 (0.5)	3 (0.1)	0.08

Frequency of detection of protozoa, helminths and bacteria in stool samples submitted by study participants each month. Months of observation were categorised as with or without diarrhoea based on recall of symptoms every two weeks. *P *determined by Fisher's exact test. ns, not significant.

**Figure 2:**
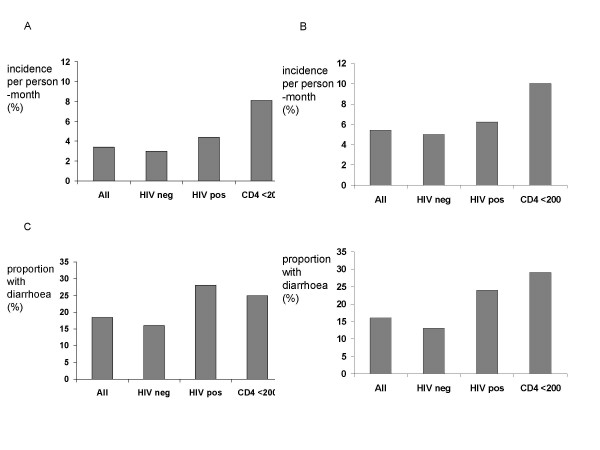
**Risk of intestinal infection and diarrhoeal disease in the whole cohort, and according to HIV status and CD4 count**. Fig 2A shows the absolute risk of intestinal infection per month, irrespective of symptoms, for pathogens using the restrictive definition (see Methods), and Fig 2B shows the absolute risk of infection per month, irrespective of symptoms, using the more open definition. Fig 2C shows the percentage of intestinal infections associated with diarrhoea (i.e. disease-to-infection ratio) using the restrictive definition. Fig 2D shows the disease-to-infection ratio using the more open definition.

The overall risk of intestinal infection for pathogens associated with diarrhoea (Fig 2A) and other potential diarrhoeal pathogens (Fig 2B) was higher only in those with low CD4 counts. However the disease-to-infection ratio was elevated for HIV seropositive participants irrespective of CD4 count whether using the restrictive definition (Fig 2C) or the more open definition of pathogen (Fig 2D).

### Clustering, susceptibility and resistance

Clustering of incidence (i.e. aggregation) was analysed in 192 participants, as those with under 10 months of observation were excluded. Diarrhoea incidence was over-dispersed in both HIV seropositive (α = 0.263; p < 0.0001) and HIV seronegative adults (α = 0.983; p < 0.0001), and in adults with CD4 counts of less than 200 cells/μL (α = 0.48; p = 0.001). In a negative binomial model the diarrhoea incidence rate ratio for HIV infection was 2.4 (95%CI 1.7–3.3; p < 0.001).

At an individual level the degree of clustering could be quite pronounced, with some adults experiencing frequent diarrhoea. Conversely, within the group of HIV seropositive participants, certain individuals were apparently resistant to diarrhoea. These included a 34-year old woman with a CD4 count of 52 who experienced no diarrhoea at all in 26 months of follow-up. Also a 29-year old woman with a CD4 count of 109 who experienced no diarrhoea in 36 months of follow up. Three other women (CD4 counts of 399, 444 and 451) experienced no diarrhoea at all.

Allowing for the clustering of diarrhoea in individuals, the incidence of diarrhoea was related independently to the presence of HIV infection (IRR 2.2, 95%CI 1.5–3.2; p < 0.001), not to immunosuppression *per se*, and to the housing hygiene index below the median (IRR 1.7, 95%CI 1.2–2.4; p = 0.002). There was no evidence of clustering of diarrhoea by household co-habitation nor by genetic relatedness. Diarrhoea incidence was not reduced in families which reported that they boil water.

Conversely, there was no evidence of clustering of infection with pathogens, either using the restrictive definition (α = 10^-14^; p = 1.0) or the more open definition (α = 10^-11^; p = 1.0).

## Discussion

Although many studies of diarrhoeal disease in AIDS in Africa were done in the pre-HAART era, there is little community-based information on the changes in susceptibility to intestinal infections and diarrhoea in relation to stage of HIV disease. The data presented here indicate that adults living with HIV/AIDS in the pre-HAART era were at increased risk of diarrhoeal disease, with a rate ratio of 2.4. This increased risk was not confined to adults with low CD4 count, and was predominantly due to an increase in the disease-to-infection ratio at all stages of HIV infection. There was also an increase in the risk of infections, whether or not associated with diarrhoea, when the CD4 count fell below 200 cells/μL. We have previously shown that it is predominantly in this latter group that nutritional impairment is found [31], and it is in this group that enteropathy is predominantly found [5]. We conclude that HIV leads to T cell dysfunction even in its earliest stages, leading to increased risk of diarrhoea when infections occur, but the increased susceptibility to infection occurs later in the course of HIV infection, which is the time when enteropathy supervenes. Taken together, our current and previous studies [5,18] suggest that enteropathy in Zambian adults is a consequence of the increased susceptibility to intestinal infection which occurs in the later stages of HIV infection, and is therefore unlikely to be a driver of HIV disease progression in the earlier stages as Douek's hypothesis would suggest [13].

In this study, there was clear evidence of clustering (i.e. aggregation) of diarrhoeal disease over time in certain individuals, indicating that some people are more susceptible to diarrhoea than others. This confirms and extends observations made before the HIV pandemic [10-12]. Interestingly, this aggregation of disease is still seen in severely immunosuppressed adults with CD4 counts of less than 200 cells/μL, so it seems unlikely to be attributable to variations in cell-mediated immunity. Humoral (antibody-mediated) immunity does not seem to play a major role in defence against intracellular pathogens (such as *C. parvum, I. belli*, or the microsporidia), so we postulate that susceptibility and resistance are determined by variations in innate immunity, possibly genetic. We were unable to demonstrate clustering of incidence rates in first-degree relatives in this cohort, but genetic studies would have to be much larger to have the power to dissect out the influence of genetic variation. While we observed clustering of incidence of diarrhoea over time in susceptible individuals, this was not observed for intestinal infection with potential pathogens. This probably means that the increased incidence of diarrhoea in these susceptible adults is not due to an increased risk of colonisation. We are then left to postulate that it is due to an inability to control the intensity of infection (leading to increased disease-to-infection ratio) or to prevent the expression of virulence factors.

In a study of this nature, ascertainment of symptoms may be a problem, even when the study cohort is under close supervision as it was in our study. We recruited participants from a small number of houses on one contiguous area within Misisi township, a very disadvantaged residential area, for two reasons. The first reason was to approximate to the assumption of uniform exposure to diarrhoea-causing organisms. The second reason was to ensure effective follow-up of the participants so that disclosure of symptoms would be as full as possible. While we cannot be sure that every episode of diarrhoea was recorded, we have only analysed months of follow-up in which a given participant was interviewed and asked directly about whether they had experienced diarrhoea in the previous two weeks. Our reliance on reported symptoms of diarrhoea also introduces the difficulty of ascertaining when episodes become persistent (i.e. over 14 days duration). While some cases were clearly persistent, it can be difficult to distinguish clinically between variation in symptoms and new episodes of diarrhoea, and it is not clear if the definition used in children (3 days [10]) applies in HIV infection. Most episodes of diarrhoea were clearly not persistent, and even cryptosporidiosis, which is strongly associated with persistence, was persistent in at most 11% of incident episodes. One further potential problem arises from the timing of sample collection. It is possible that symptoms could have been reported at the end of a month and the infection only discovered in the next sample submitted at the beginning of the following month. By recording symptoms every two weeks we tried to minimise this problem, but it cannot be avoided altogether and would have the effect of making the association between symptoms and infections weaker than is really the case. It would also reduce the estimated disease:infection ratio but the difference between HIV infected and uninfected episodes should still be measurable, as indeed we found.

The assumption that exposure was intense and consistent in this community underlies our analysis. Infecting dose has an important influence on clinical manifestations of intestinal infectious disease [32,33] so intensity of exposure could determine disease-to-infection ratio and susceptibility. If behavioural factors did play a major role in determining the incidence of diarrhoea, we might expect to observe some clustering of incidence in households, but this was not observed. We were also unable to detect any influence of boiling of drinking water on diarrhoea incidence, but as we did not verify the statements made by respondents against actual observable practices, it is possible that a small effect may have been missed.

The profile of infections seen in our participants is typical of the spectrum of pathogens which are seen in African AIDS patients [18,34], with a preponderance of protozoa. We did not test for viruses, but we do know that rotavirus is an extremely rare cause of diarrhoea in Zambian AIDS patients (P. Kelly, unpublished observations). We have previously reported the high frequency of isolation of *Citrobacter rodentium *[5].

In this population with an HIV seroprevalence of 31% and in which 10% of all adults were immunocompromised, the attributable risk of diarrhoea due to HIV (risk in HIV seropositives minus risk in HIV seronegatives) was 0.312 episodes per year. The population attributable risk fraction is therefore 28%. It seems likely that the ongoing expansion of access to anti-retroviral drugs will ameliorate the increased susceptibility to infection and to disease in similarly affected populations. In view of the high proportion of asymptomatic infections at all CD4 counts, however, it seems unlikely that improved cell mediated immunity in HIV-infected patients treated with anti-retroviral drugs will have a significant impact on the transmission of diarrhoeal pathogens from asymptomatic adults to other adults and to children. Improved water quality, sanitation and food hygiene will remain high priorities for families caring for HIV-infected adults for the foreseeable future.

## Conclusion

HIV infection is associated with an increased disease-to-infection ratio at all stages of infection and with increased susceptibility to infection as CD4 count declines. Diarrhoeal disease is aggregated in susceptible individuals irrespective of CD4 count suggesting that it is not a function of cell mediated immunity.

## Abbreviations

HIV: Human Immunodeficiency Virus; HAART: Highly Active Anti-Retroviral Therapy; CD4: Cluster Differentiation Antigen 4; IRR: Incidence Rate Ratio.

## Competing interests

The authors declare that they have no competing interests.

## Authors' contributions

PK, SS, JM, MJF and RAF designed the study. PK, JT, SS, HS and MK analysed the samples and the data. PK and JT wrote the manuscript, and all authors revised and checked it.

## Pre-publication history

The pre-publication history for this paper can be accessed here:

http://www.biomedcentral.com/1471-230X/9/7/prepub
